# Supplementary material for: Late Holocene slowdown of the Indian Ocean Walker circulation
Source: Nat Commun. 2017 Oct 18;8:1015. doi: 10.1038/s41467-017-00855-3 (PMC5715104; doi:10.1038/s41467-017-00855-3)
Supplement: Supplementary file 1 — Supplementary Information [file 41467_2017_855_MOESM1_ESM.pdf]

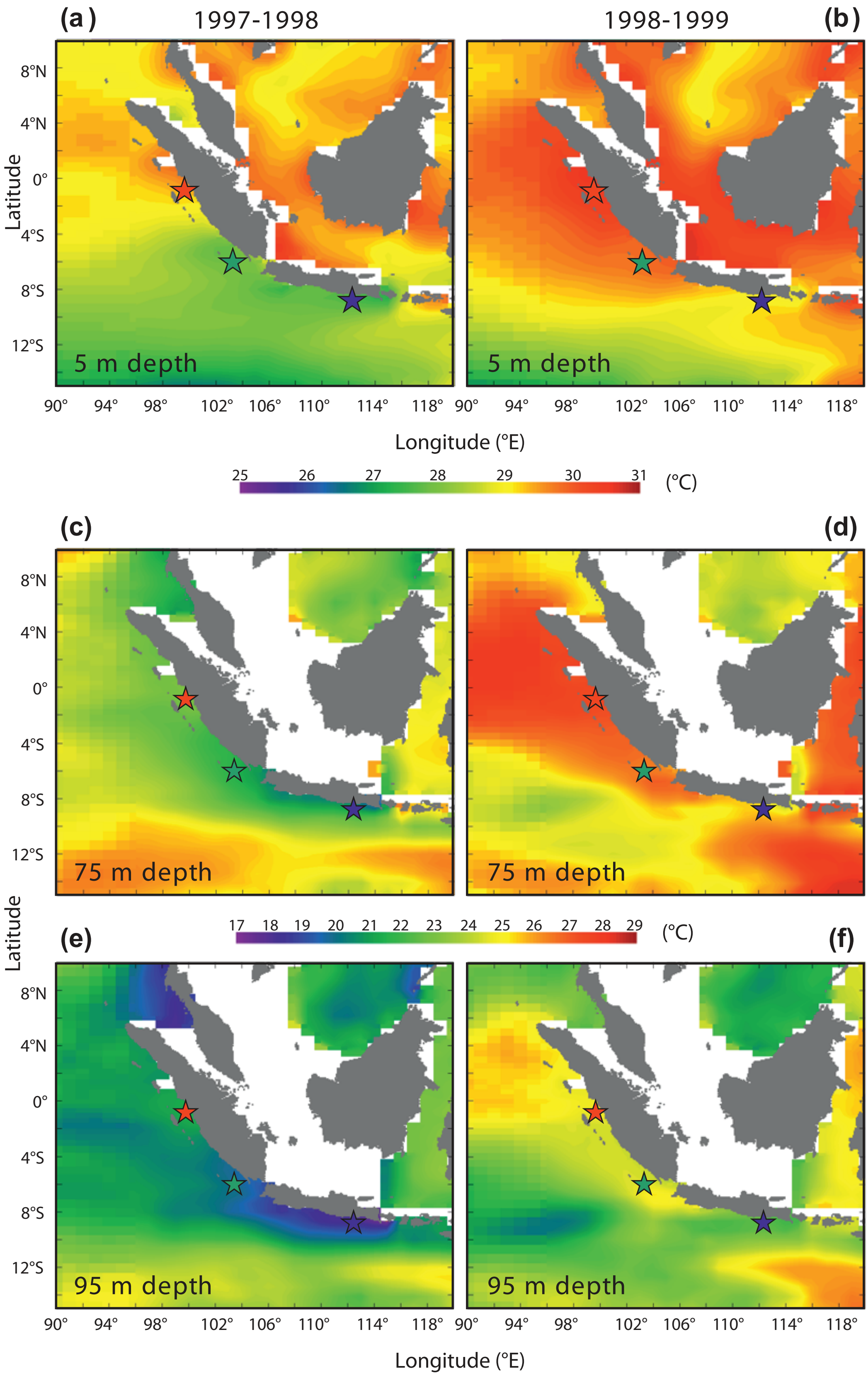
(a)
1997-1998
(b)
1998-1999
8°N
4°N
0°
4°S
8°S
12°S
Latitude
5 m depth
5 m depth
90° 94° 98° 102° 106° 110° 114° 118°
90° 94° 98° 102° 106° 110° 114° 118°
Longitude (°E)
25 26 27 28 29 30 31 (°C)
(c)
(d)
75 m depth
75 m depth
17 18 19 20 21 22 23 24 25 26 27 28 29 (°C)
(e)
(f)
95 m depth
95 m depth

Supplementary Figure 1. Modern changes in mean annual surface and thermocline temperatures of the eastern tropical Indian Ocean. Potential temperature at **(a, b)** 5 m water depth, **(c, d)** 75 m water depth, and **(e, f)** at 95 m water depth during a positive Indian Ocean Dipole - El Niño year from March 16, 1997 to March 16, 1998 **(a, c, e)** and a La Niña year from March 16, 1998 to March 16, 1999 **(b, d, f)**. Note the different scale for **(a, b)**. Data from http://data1.gfdl.noaa.gov. Stars indicate the position of the records presented in this study (red: 39KL, green: GeoB 10038-4, blue: GeoB 10053-7). Note the observed warming in a strong Walker circulation scenario (right) compared to a weak Walker circulation scenario (left).

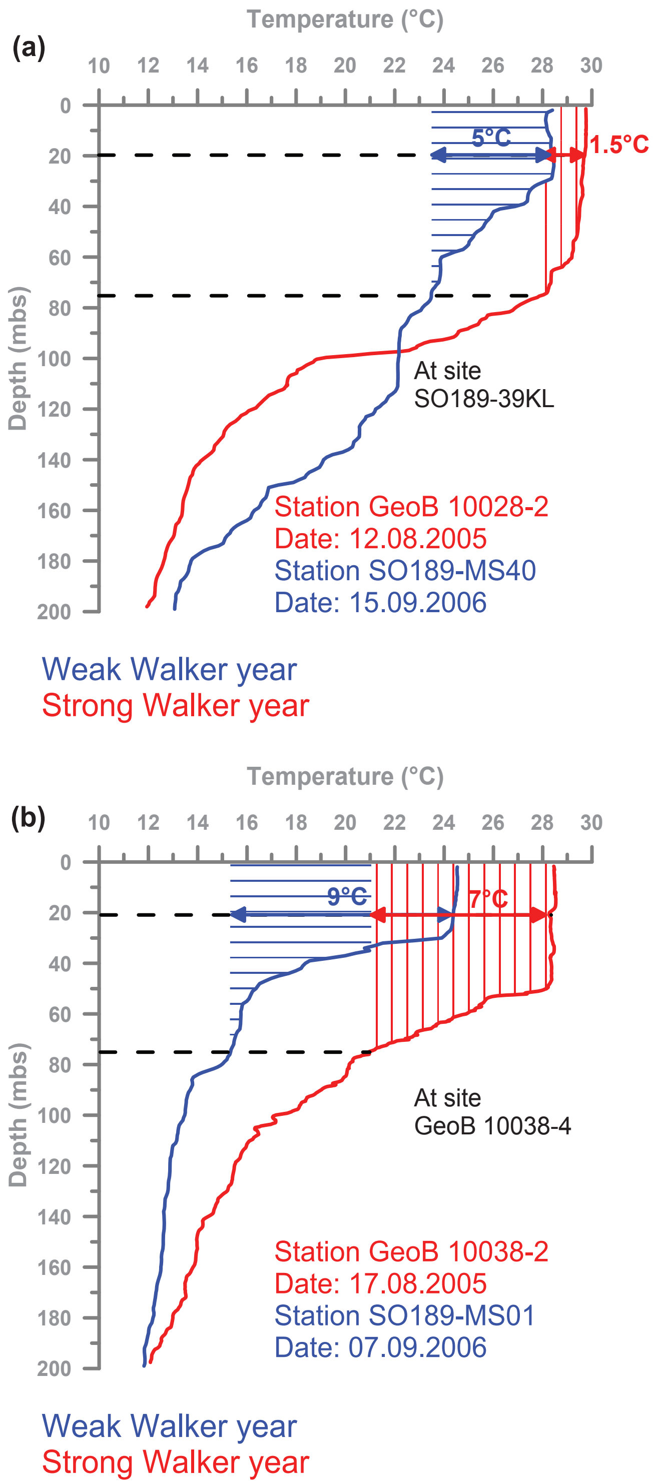

Supplementary Figure 2. Response of the water column structure in the eastern tropical Indian Ocean to changes in Walker circulation. CTD data from a strong Walker circulation year (2005, red) and a weak Walker circulation year (2006, blue) at sites **(a)** SO189-39KL and **(b)** GeoB 10038-4. Note the deeper and warmer thermocline during the strong Walker year. Double-headed arrows and coloured strips show the temperature difference (T) between 20 m and 75 m water depth indicated by dashed lines. Note the smaller T during the strong Walker year.

(a)

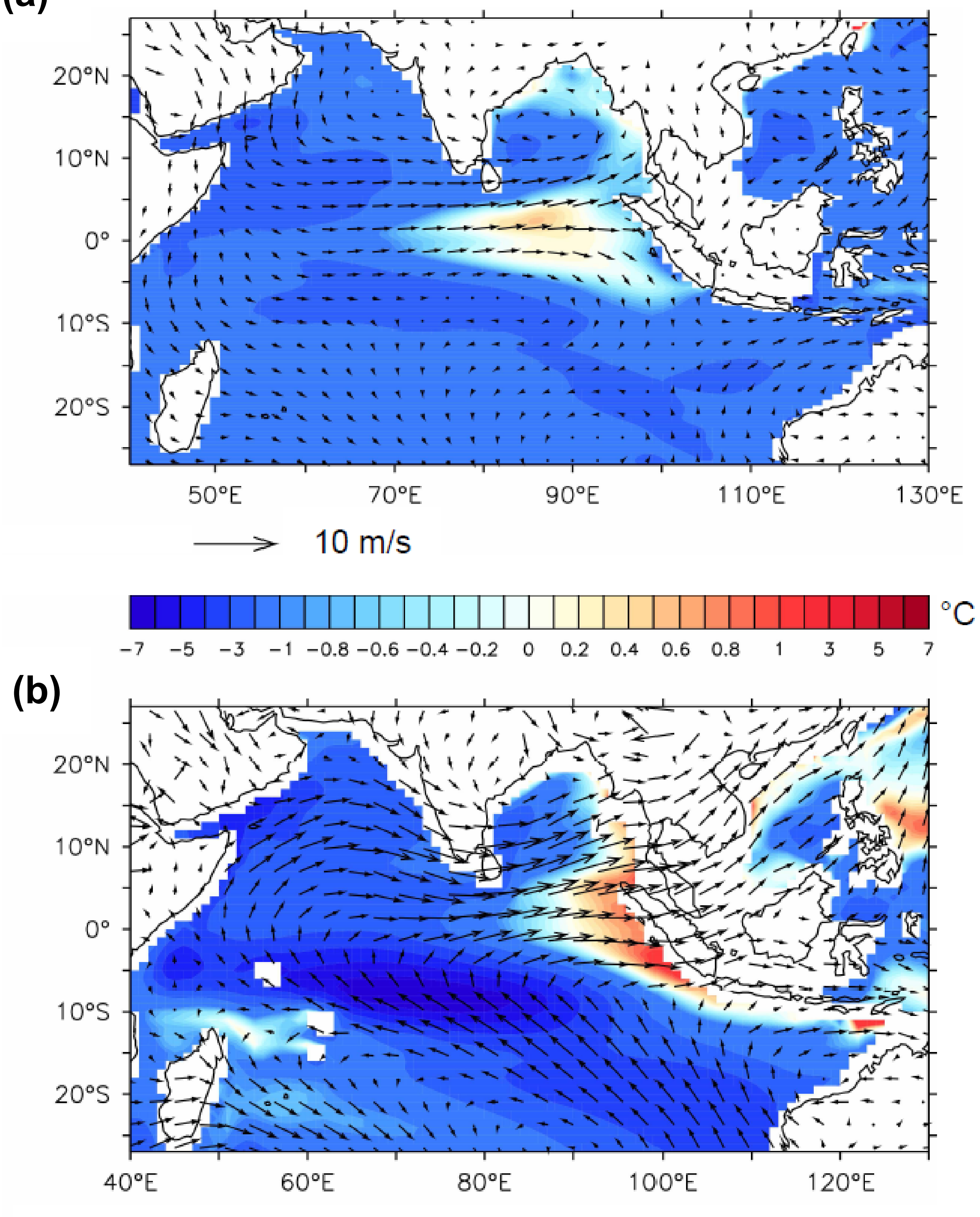

Supplementary Figure 3. LGM ocean subsurface warming in CCSM3 and FGOALS-g1.0. Shown are LGM anomalies (annual mean) relative to pre-industrial control runs for the 70 m temperature for **(a)** CCSM3 and **(b)** FGOALS-g1.0. Low-level wind anomalies and coastlines as in Fig. 5.

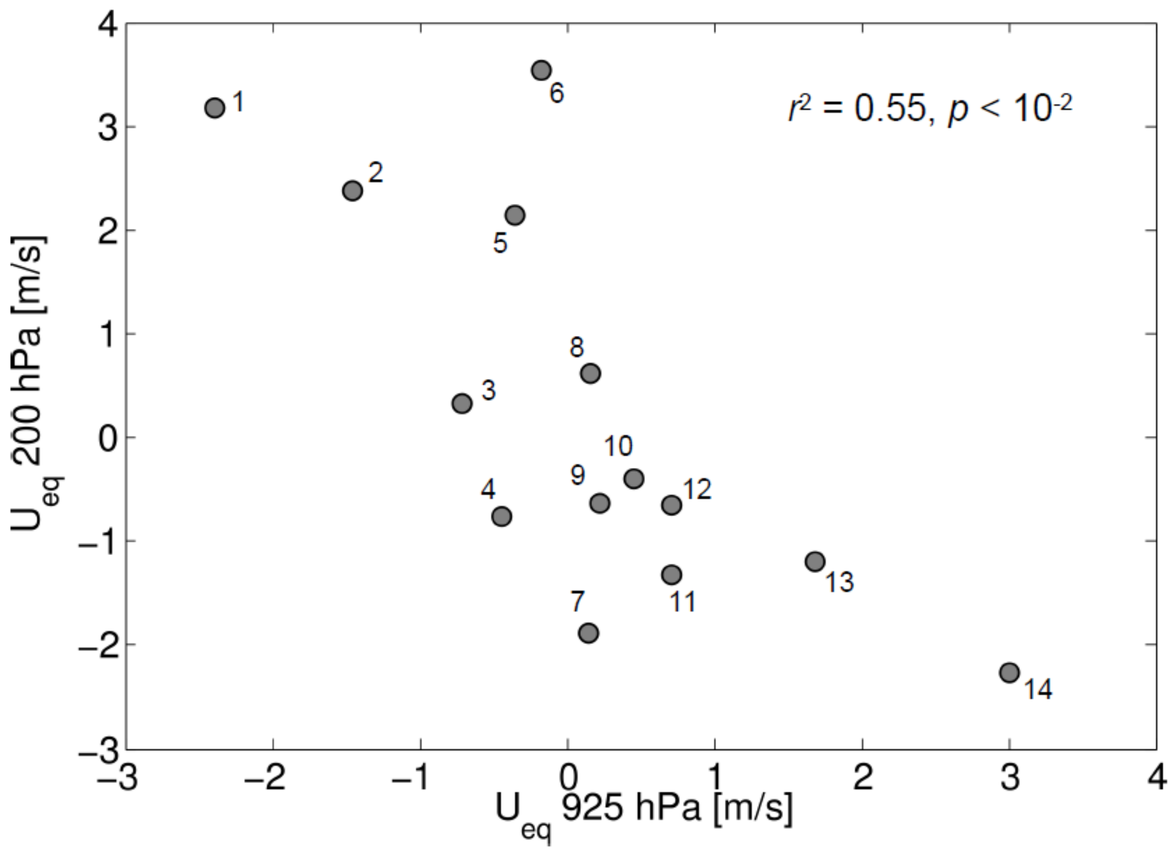

Supplementary Figure 4. Upper-tropospheric (200 hPa) zonal wind anomalies versus low-level (925 hPa) zonal wind anomalies over the equatorial Indian Ocean as simulated in PMIP2 and PMIP3/CMIP5 models. Zonal wind anomalies, U_{eq}, are defined as the annual mean zonal wind averaged over 5°N-5°S, 50°E-100°E (see Fig. 5), LGM relative to pre-industrial. The models are numbered as in Supplementary Table 2.

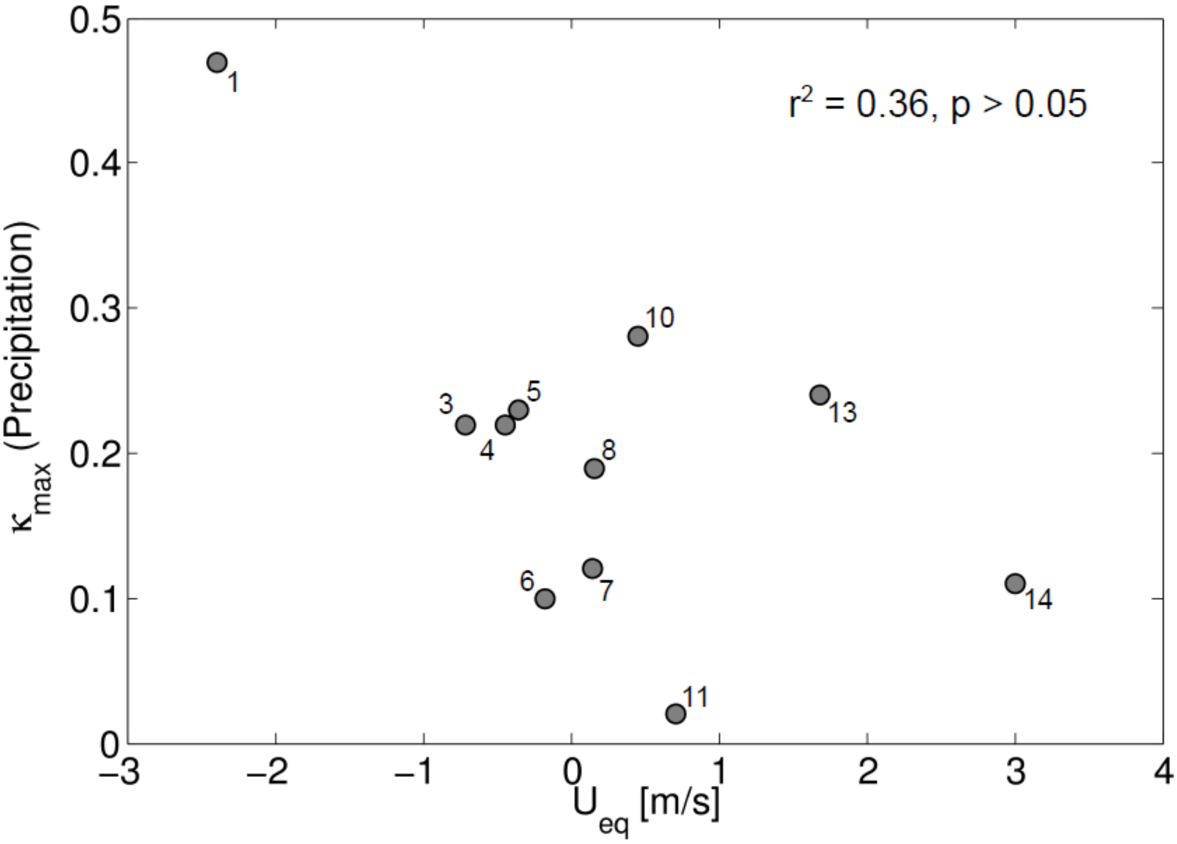

Supplementary Figure 5. Maximum Cohen's κ versus equatorial Indian Ocean zonal wind anomaly as simulated in PMIP models. U_{eq} is defined as the LGM anomaly (relative to pre-industrial) of the annual mean zonal low-level (925 hPa) wind averaged over 5°N-5°S, 50°E-100°E (see Fig. 5). Maximum Cohen's κ has been used to quantify model-data agreement with respect to precipitation proxies in the Indo-Pacific region, for the calculation of Cohen's κ see[1]. The models are numbered as in Supplementary Table 2. Three models (No. 2, 9, and 12) were not included in (ref [1]), hence no κ values are available.

Core/Species	# Samples	# Replicates	1σ Standard Deviation (mmol mol^{-1})		
			Ext. standard	ECRM 752-1	Replicates
**SO189-39KL**					
G. ruber	671	55	0.005	0.006	0.12
P. obliquiloculata	651	50	0.004	0.003	0.10
**GeoB 10038-4**					
G. ruber	184	39	0.09	0.009	0.09
P. obliquiloculata	60	26	0.09	0.009	0.22
N. dutertrei	248	94	0.09	0.009	0.25
G. tumida	62	25	0.09	0.009	0.11
**GeoB 10053-7**					
G. ruber	291	30	0.01	0.01	0.19
P. obliquiloculata	271	30	0.02	0.01	0.21

Supplementary Table 1. Amount and relative standard deviation of Mg/Ca measurements at different sites. Standard deviations (1σ) for external standard and ECRM 752-1 are calculated by considering all measurements during a analytical campaign as indicated in the column "# Samples". Standard deviation for replicates refers to measurements on additional samples that have been separately cleaned and measured (column "# Replicates"), and then compared to their corresponding samples from the original run (column # Samples).

No.	Model	Institution	Grid resolution	PMIP phase
1	HadCM3	UK Met Office Hadley Centre, UK	Atm: 96 x 72 x L19<br>Ocn: 288 x 144 x L20	2
2	FGOALS-g2.0	LASG/Institute of Atmospheric Physics, China	Atm: 128 x 60 x L26<br>Ocn: 360 x 180 x L30	3
3	IPSL-CM4	Institut Pierre Simon Laplace, France	Atm: 96 x 72 x L19<br>Ocn: 182 x 149 x L31	2
4	CNRM-CM5	Centre National de Recherches Meteorologiques, France	Atm: 256 x 128 x L31<br>Ocn: 362 x 292 x L42	3
5	GISS-E2-R	NASA Goddard Institute for Space Studies, USA	Atm: 144 x 90 x L40<br>Ocn: 288 x 180 x L32	3
6	IPSL-CM5A	Institut Pierre Simon Laplace, France	Atm: 96 x 96 x L39<br>Ocn: 182 x 149 x L31	3
7	MRI-CGCM3	Meteorological Research Institute, Japan	Atm: 320 x 160 x L48<br>Ocn: 364 x 368 x L51	3
8	MIROC3.2	Center for Climate System Research (University of Tokyo), JAMSTEC, Japan	Atm: 128 x 64 x L20<br>Ocn: 256 x 192 x L43	2
9	ECHAM5-MPIOM	Max Planck Institute for Meteorology, Germany	Atm: 96 x 48 x L19<br>Ocn: 120 x 101 x L40	2
10	MPI-ESM	Max Planck Institute for Meteorology, Germany	Atm: 196 x 98 x L47<br>Ocn: 256 x 220 x L40	3
11	CCSM4	National Center for Atmospheric Research, USA	Atm: 288 x 192 x L26<br>Ocn: 320 x 384 x L60	3
12	MIROC-ESM	JAMSTEC, University of Tokyo, National Inst. for Environmental Studies, Japan	Atm: 128 x 64 x L80<br>Ocn: 256 x 192 x L44	3
13	CCSM3	National Center for Atmospheric Research, USA	Atm: 128 x 64 x L26<br>Ocn: 320 x 384 x L40	2
14	FGOALS-g1.0	LASG/Institute of Atmospheric Physics, China	Atm: 128 x 60 x L26<br>Ocn: 360 x 180 x L33	2

Supplementary Table 2. PMIP2 and PMIP3/CMIP5 models providing LGM and pre-industrial simulations used in this study. In the column for grid resolution the following abbreviations are used: Atm (atmospheric grid resolution), Ocn (ocean grid resolution), L (number of levels in the vertical). The numbering (first column) is related to the simulated magnitude of LGM zonal wind anomalies over the equatorial Indian Ocean and used in Fig. 4 and Supplementary Figs. 4-5. For further references regarding the climate models the reader is referred to https://pmip2.lsce.ipsl.fr and https://pmip3.lsce.ipsl.fr.

Supplementary references

1 DiNezio, P. N. & Tierney, J. E. The effect of sea level on glacial Indo-Pacific climate. *Nature Geoscience* **6**, 485-491, doi:10.1038/ngeo1823 (2013).
