# Supplementary material for: Late Holocene slowdown of the Indian Ocean Walker circulation
Source: Nat Commun. 2017 Oct 18;8:1015. doi: 10.1038/s41467-017-00855-3 (PMC5715104; doi:10.1038/s41467-017-00855-3)
Supplement: Supplementary file 3 — Description of Additional Supplementary Files [file 41467_2017_855_MOESM3_ESM.pdf]

Description of Additional Supplementary Files

File Name: Supplementary Data 1
Description: Hydroclimate proxy data from the eastern tropical Indian Ocean. This file contains Mg/Ca-based SST and thermocline temperatures with 1σ error at different sites, their difference (ΔT), and the average ΔT values for different periods with 95% confidence interval (CI). Also shown are hydrogen isotope (δD) values of n-C_{31} alkanes relative to the standard mean ocean water (‰ SMOW) with the 95% CI (LGM values are corrected for ice-volume), the C_{31} n-alkane concentrations in nanogram per gram sediment (ng g^{-1}), the Carbon Preference Index (CPI_{27-33}) as a measure of the degree of terrestrial organic matter degradation (higher CPI reflects less degraded plant waxes and vice versa), and their 1σ standard deviations (SD).
